# Non-Viral Vectors for Delivery of Nucleic Acid Therapies for Cancer

**DOI:** 10.3390/biotech11010006

**Published:** 2022-03-07

**Authors:** Shrey Kanvinde, Tanmay Kulkarni, Suyash Deodhar, Deep Bhattacharya, Aneesha Dasgupta

**Affiliations:** 1 Department of Pharmaceutical Sciences, University of Nebraska Medical Center, Omaha, NE 68198, USA; tkulkarni68@gmail.com (T.K.); deep.bhattacharya1991@gmail.com (D.B.); 2 Department of Pharmacology and Experimental Neuroscience, University of Nebraska Medical Center, Omaha, NE 68198, USA; suyashsanjay.deodhar@unmc.edu; 3 Department of Biochemistry and Molecular Biology, Mayo Clinic, Rochester, MN 55905, USA; dasgupta.aneesha@mayo.edu

**Keywords:** gene therapy, non-viral vectors, cancer, nucleic acid delivery, nanoparticles, polymers, lipids, lipid nanoparticles, mRNA, siRNA, regulatory landscape, translation, CMC

## Abstract

The research and development of non-viral gene therapy has been extensive over the past decade and has received a big push thanks to the recent successful approval of non-viral nucleic acid therapy products. Despite these developments, nucleic acid therapy applications in cancer have been limited. One of the main causes of this has been the imbalance in development of delivery vectors as compared with sophisticated nucleic acid payloads, such as siRNA, mRNA, etc. This paper reviews non-viral vectors that can be used to deliver nucleic acids for cancer treatment. It discusses various types of vectors and highlights their current applications. Additionally, it discusses a perspective on the current regulatory landscape to facilitate the commercial translation of gene therapy.

## 1. Background

Development of new therapies for cancer represents one of the most significant challenges in medicine. Despite significant progress in understanding the underlying mechanisms of cancer progression, there has not been a significant number of successful commercial therapies. The fiscal budget of the National Cancer Institute for 2022 mentions an approximate 20% increase in the cancer research budget, and a 50% increase in grants submitted, which has resulted in a dramatic increase in survival rates over the last decade [1]. The NCI projects about 22 million cancer survivors by 2030. Despite this, the 5-year survival rates of pancreatic cancer (9%), lung cancer (7%), liver cancer (20%), and glioblastoma (5%) are at a dismal low. Even cancers which are responsive to treatments and have higher 5-year survival rates, such as breast cancer (90%), have low treatment efficacies at later stages, leading to about 45,000 deaths in the US in 2021 (American Cancer Society. Cancer Facts and Figures 2021). Current non-invasive therapies utilize chemotherapeutic agents or radiation, which usually results in cytotoxicity to other cells of the host body.

Genetic mutation or genome instability is an important hallmark of cancer. Mutations can be genetic or can arise spontaneously due to environmental factors. There are two kinds of genes which contain mutations—proto-oncogenes and tumor-suppressor genes (TSGs) [2]. Mutations in proto-oncogenes are mainly gain of function, which lead to enhance proliferation while those in TSGs are loss of function, which lead to decreased cell death. The mutator phenotype hypothesis states that, since normal mutation rates are insufficient to explain the large number of mutations, cancer cells possess an increased rate of mutagenesis [3]. Mutations can occur from DNA damage or from misincorporation by DNA polymerases. These mutations are called “drivers” for their ability to progress tumorigenesis. In the past few decades, it has been reported that cancer genomes contain many additional “passenger” mutations [4]. Mutations in cancer lead to insights of their manifestation as a disease of genetic mutations.

Based on the reported genetic involvement in cancer, gene therapy (GT) was proposed as a promising field, which was speculated to usher in a new era of treatments. Of mention are various novel nucleic acid (NA) gene therapies, such as plasmid (pDNA), messenger RNA (mRNA), oligonucleotides, and RNA interference strategies (siRNA, shRNA, miRNA) (Table 1), with the latter being the topic that led to Andrew Fire and Craig Mello receiving the Nobel Prize in Physiology or Medicine in 2006 [5]. GTs exert their therapeutic actions may be by excising or silencing pathological genes, increasing expression of key proteins in metabolic pathways, or through targeted sequestration of proteins, cofactors, or mRNA. While pDNA remains the cornerstone of classical gene therapy, its use has been reduced to the advent of better avenues. mRNA-based therapies have expanded beyond protein replacement and gene replacement in cancer treatment, and have shown a meteoric rise in vaccine development against SARS-CoV-2 [6]. Additionally, mRNA therapeutics have an added advantage of better safety profile and faster protein expression compared with pDNA therapeutics, since an integration of the nucleic acid cargo into the host genome is not required for expression [7]. Efforts focused on harnessing the intrinsic properties of nucleic acid drugs has led to the development of many clinically deployed nucleic-acid-based therapeutics, such as aptamer RNAs (e.g., pegaptanib), antisense oligonucleotides (ASOs), or antisense RNAs (asRNAs) (e.g., mipomersen, eteplirsen, nusinersen, inotersen, and golodirsen), and short interfering RNAs (siRNAs) (e.g., patisiran and givosiran).

In the past three decades, approximately 2000 GT clinical trials have been conducted, two-thirds of which have been in cancer therapy [8]. Cancer GTs can include targeting various aspects of tumor biology, such as silencing oncogenes, tumor-suppressor enhancement, mutation correction, and stimulation of immune cells against the tumor [9]. While the advancements made in the nucleic acid cargo have been substantial, the present challenges predominantly lie with the specific and efficient delivery of these therapeutics [10]. Free nucleotides and nucleic acids are large macromolecules that are rapidly degraded in biological microenvironments and body fluids. Secondly, owing to their bulky structure and negative charge, they have poor in vivo biodistribution and penetration across biological and cell membrane barriers. Lastly, induction of rapid immune response to sequester and subsequently degrade nucleic acids in extracellular environment also reduces the accumulation and delivery of nucleic acids to target tissues. All these factors necessitate the need of an efficient delivery system for nucleic acid payloads. An ideal delivery system must encapsulate the nucleic acid cargo, protect it from degradation, retain the macromolecular conformation of the cargo, and deliver it to a selective target tissue, as well as deliver it within the appropriate cellular compartment [11]. Unfortunately, significant challenges with the development of these gene delivery vectors have resulted in an unsuccessful clinical and commercial translation. However, thanks to development of new nucleic acid therapeutic payloads, as well as novel delivery vehicles, there has been a revival of the promise of GT in cancer treatment.

## 2. NA Delivery Vectors

Gene delivery vectors can be classified into viral and non-viral vectors. Viral vectors involve replacing the virus genome with the gene of interest and then using the virus, such as the particle, to transfect at the target site [12]. Viral vectors are starting to emerge in the field; however, more work in terms of formulation and process development needs to be carried out [13]. In the past, even though viral vectors have been shown to be stable and can withstand high temperatures, they do possess some inherent challenges in gene delivery [14]. However, the main drawbacks of using viral vectors are their immunogenicity and cytotoxicity. Additionally, a very impactful element to mention is a concern known as insertional mutagenesis, which is the ectopic chromosomal integration of viral DNA [15]. This can lead to the inhibition of tumor-suppressor gene expression or the expression and activation of oncogenes, leading to a malignant transformation of the cells. Additionally, human studies have also shown a likelihood of immune response. This generation of neutralizing antibodies in response to viral vectors has been well illustrated in Hemophilia B trials [16]. Table 2 summarizes the different types of viral vectors along with their advantages and limitations.

Other than viral vectors, delivery methods have also been used for successful nucleic acid delivery into cells. Delivery methods include physical methods that can enhance the delivery of nucleic acids through some physical stimulus. Physical delivery methods—although not the focus of this manuscript—form an important tool in the arsenal of nucleic acid delivery. Table 3 elucidates various physical methods of nucleic acid delivery along with their mechanism, preferred site of action, advantages, and limitations.

Physical methods of nucleic acid transfer are limited to DNA applications. Structurally, DNA is a much more robust as compared with novel payloads, such as siRNA, mRNA, etc. As a result, significant amount of optimization work would be needed to adapt these methods to delivery novel nucleic acid payloads.

In contrast, non-viral vectors can overcome the immunogenicity and cytotoxicity concerns observed with viral vectors [15]. Reduced pathogenicity, in addition to low cost and ease of large-scale production, make non-viral vectors attractive gene delivery vehicles. Additionally, a combination of physical methods and non-viral vectors would represent an attractive avenue in developing new cancer therapies. In this review, we focus on elucidating various types of non-viral vectors utilized in gene delivery. For the ease of explanation, we have divided the vectors into carbohydrate-based, polymer-based, and lipid-based vectors. To conclude the review, we also touch base on the regulatory landscape and translational perspective of these vectors for gene delivery.

## 3. Carbohydrate-Based NA Vehicles

Carbohydrates and complex polysaccharides, such as cyclodextrin (CD) and chitosan, have been investigated for gene delivery so far. Polysaccharides possess oligoamine groups which are weakly cationic and can be leveraged for limited nucleic acid complexation [17]. However, the oligoamine groups can also be used as anchoring points to attach polycations. There have been multiple reports of biodegradable polycations based on grafted oligoamine residues of polysaccharides. These modifications have resulted in nucleic-acid-delivery vehicles that are biodegradable, as well as able to exhibit high transfection efficiency. In addition, polysaccharide-based nanoparticles and their subsequent use with targeting agents has exponentially increased exploration of these delivery vehicles. In this section, we go over few carbohydrate- and polysaccharide-based nucleic-acid-delivery vectors, including their advanced applications.

### 3.1. Cyclodextrin Based Vectors

Cyclodextrins (CD) are naturally occurring cyclic oligosaccharides. Due to their structure, CDs possess hydrophilic cavity exteriors and hydrophobic interiors. CDs have been explored in gene delivery due to multiple benefits. One of the early appealing benefits was the ability of CDs to reduce cytotoxicity of polycations. Hwang and co-workers synthesized a polyamidines linked with CDs [18]. They reported that the IC50 of CD-linked monomers was significantly higher than that of polyamidines lacking CDs in their structure. Such applications also gave rise to exploratory studies, where CDs could be made more amenable to gene delivery by conjugation with polycations.

CD has been chemically conjugated to polyamidoamine (PAMAM) to generate dendrimers that are highly branched three-dimensional structures around the central core (α-CD-PAMAM) [19]. This enabled the system to possess terminal basic amino groups which could form complexes with the nucleic acids while reducing cellular toxicity [20]. It was observed that α-CD-PAMAM improved the transfection while protecting the siRNA from serum nucleases. Moreover, the conjugated structures were present in cytoplasm as opposed to being present in both nucleus and cytoplasm, as in the case of control transfecting agents (such as LipofectamineTM2000 (L2000), RNAiFectTM (RF), and linear PEI) [21]. Later generation of complexes looked at utilizing targeting ligands, cell penetration enhancers, endosomal disruption agents to bestow enhanced site specificity, cell membrane penetration, and endosomal disruption, respectively. α-CD-PAMAM_G3_, with four degrees of folate substitution, were reported to exhibit the highest intracellular delivery of siRNA in folate responsive KB cells when screened in vitro and in vivo [22]. CD conjugated poly(L-lysine) dendrimer has also been reported in gene delivery to enhance cell permeation. Poly(L-lysine) mimics cell penetrating peptides, increases membrane penetration, and improves gene delivery. This CD–dendrimer system has been reported to deliver docetaxel (DOC) and matrix metalloproteinase-9 (MMP-9) siRNA. A reduction in MMP-9 levels in HNE-1 cells and higher apoptosis than DOC or plasmid individually was observed [23].

Additionally, CD-Poly(L-lysine) dendrimers conjugated with endosomal disrupting agents, such as fusogenic peptides, histidine, chloroquine, etc., have been shown to further improve the transfection efficiency of siRNA [24]. Transferrin receptors are upregulated on cancer cells to harvest iron and hence are an attractive target for gene delivery [25]. To leverage this overexpression, positively charged, CD-containing polymer (CDP) nanoplexes with nucleic acid have been reported. Transferrin was conjugated to CDP via PEG linker attached to adamantine inserted in CD cavity [26]. These nanoparticles have been shown to significantly inhibit tumor growth in a disseminated model of Ewing’s sarcoma.

Hyaluronic acid (HA) is another targeting ligand extensively used in tumor targeting since it attached to CD44 receptors overexpressed on cancer cells [27,28,29]. HA-PEI-CD complex showed significant improvement in transfection over PEI alone in three cancer cell lines (∼39.5-, 41.4-, and 8.8-fold greater in HeLa, HEK-293, and MCF-7, respectively) [30,31]. Anisamide is a targeting functional group that binds sigma receptors on cancer cells and facilitates internalization of the nanoparticle system [32]. A recently reported system utilized modified CD with guanidino group on primary hydroxyl group. This allowed complexation with VEGF siRNA, while the secondary hydroxyl group was utilized to anchor anisamide to target sigma receptor. The authors reported successful reduction in VEGF mRNA levels in PC3 prostate cancer cells and significant reduction in tumor volume in TRAMP-C1-induced xenograft mouse model of prostate cancer [33].

Sugars such as mannose and lactose are attractive ligands for targeted gene delivery via mannose, CD205, and ASGP receptors, which are overexpressed in cancer cells. Second generation of lactosylated α-CD dendrimer (Lac-α-CD-PAMAM_G2_) with 2.6 degree of substitution was found to be optimal in terms delivering pDNA in HepG2 cells via ASGP receptor [34]. Additionally, glucuronyl-glucose-modified, second-generation β-CD dendrimer (GUG-β-CD-PAMAM_G2_) with a 1.8 degree of substitution showcased high transfection ability (pDNA) in vitro (A549, RAW264.7) as well as in vivo [35]. Folic acid decorated nanoparticles latch on to tumor cells since many cancers overexpress folate receptors and hence are used as targeting ligands for gene delivery [36]. A nanoparticle system consisting of PEI cross-linked with 2-hydroxypopyl-β-cyclodextrin (HP-β-CD) and folate improved the endocytosis of complexed VEGF siRNA, demonstrating around 90% in vitro gene silencing efficiency and reduced vascular endothelial growth factor (VEGF) protein expression [37]. In vivo studies in mice demonstrated tumor growth inhibition and reduced VEGF expression in tumors. Another CD-containing system for siRNA delivery against prostate specific membrane antigen (PSMA) was reported [38]. High uptake in two prostate cancer cell lines (LNCaP, VCaP) was observed. Apart from previously mentioned ligands, rabies virus glycoprotein and antibodies are additional modalities for targeted gene delivery in glioblastoma and blood cancer, respectively [39].

CD applications have also been reported in gene delivery vehicles that deliver cargo upon external stimulus. Within cancer applications, these stimuli are often tumor-specific environmental factors, such as pH, redox state, or over-expressed enzymes [40]. Delivery vehicles take advantage of the various pH conditions they encounter in physiological conditions, i.e., lysosomes/endosomes with acidic pH (~6.0), tumor microenvironment with slightly acidic pH (6.5–6.8), and basic pH (7.4) in healthy tissue. Common pH-responsive functions that are responsible for drug release include covalent bonds, such as hydrazine, imine, carboxyl, acetyl, cyclic orthoester, etc. [41]. A siRNA delivery system for head and neck squamous cell carcinoma was developed by incorporating a graft of three types of methacrylates attached to β-CD by acid–labile hydrazone bond [42]. The terminally located N, N, N-trimethylaminoethyl methacrylate (TMAEMA) provides positive charge responsible for siRNA complexation and forms a graft along with hydrophobic hexyl methacrylate (HMA) and pH-sensitive 2-(dimethylamino)ethyl methacrylate (DMAEMA) monomers. This system reduced mRNA and anti-apoptotic Bcl-2 protein levels in UM-SCC-17B cancer cells by 50–60% and 65–75%, respectively [43]. Tumors show a high level of glutathione (GSH) which can be harnessed to develop systems that contain redox responsive functional groups and show redox-based drug release [44]. A nanoparticle system composed of poly(β-CD) backbone and disulfide-linked poly-DMAEMA arms and auxiliary targeting system exhibited excellent pDNA transfection efficiency in KB, HeLa, COS-7 cells [24]. The same system was also reported to exhibit gene silencing using siRNA in KB_EGFP and COS_EGFP cells.

CDs also possess unique structural characteristics. CDs form a supramolecular structure called polyrotaxane (PR) [45]. These are unique molecules, where CDs are threaded and are free to rotate and slide along an axial long polymer chain that is capped at both ends by stopper groups. Upon cleavage of the stoppage groups, the CDs are released. A PR system consisting of α-CD and PEG exhibited efficient gene knockdown by siRNA delivery in comparison to branched PEI and Lipofectamine 2000 [46]. In a separate study, PRs containing 2-hydroxypropyl-β-CD with cholesterol end caps designed for siRNA delivery showed more than 80% gene silencing in vitro (3T3-GFP, HeLa-GFP) [47]. Moreover, a pH-responsive PR system for siRNA delivery was developed by threading N, N-dimethylaminoethyl (DMAE)-modified α-CD segments onto PEG capped with bulky N-benzyloxycarbonyl-L-tyrosine groups via cleavable disulfide bonds [48]. The siRNA mediated silencing of a firefly luciferase gene was successfully observed in HeLa cells [49].

### 3.2. Chitosan-Based Vectors

Chitosan (CS) is a biodegradable and biocompatible long-chain polysaccharide [50]. The primary amino groups are responsible for imparting not only positive charge in physiological conditions but also their possible versatility in terms of functionalization. Hydroxyl groups are also utilized for functionalization purposes. However, the high positive charge density of CS is a driving factor behind its use as gene delivery vector. CS is modified with a hydrophobic moiety to generate an amphiphilic polymer that can be utilized for gene delivery vector development purposes. The hydrophobic moieties aid in membrane penetration and facilitate release in cytoplasm [51].

Stearic acid (SA)-grafted CS was developed and studied for gene delivery applications [52]. The screening studies with varying MW and substitution degree of polymers indicated towards the stability of vectors made of low MW polymer. Additionally, the size of the nanoparticle/DNA complex was large when high MW CS was used with same percent substitution degree of SA as well as high substitution degree of SA with same MW of CS. High transfection efficiency was observed in A549 cells [52]. In a separate study, a trimethyl chitosan functionalized with SA by amidation of glucosamine residues through the reaction of TMC with N-succinimidyl stearate was evaluated for splice-switching oligonucleotide (SSO) delivery [53]. A high transfection efficiency was observed in HeLa/Luc705 cells. Alternatively, linoleic acid and penetratin dual-functionalized CS generated by reacting CS with linoleic acid via EDC/NHS coupling reaction followed by carbodiimide conjugation of penetratin showed efficient protection of pDNA from DNase I attack and exhibited ∼34–40-fold higher transfection in comparison with unmodified chitosan in HEK 293, CHO, and HeLa cells [54]. In a separate study, folate modified linoleic acid and poly (β-malic acid)-double-grafted chitosan nanoparticles were developed for the codelivery of paclitaxel and pDNA [55]. These nanoparticles exhibited enhanced antitumor efficacy and prolonged survival period in hepatoma-bearing mice. Quaternized N-(4-pyridinylmethyl) CS nanoparticles developed as delivery vectors for pDNA showed transfection efficiency in Huh 7 cells due to the 4-pyridinylmethyl substitution on CS, which promoted the interaction and condensation with DNA as well as N-quaternization, which increased CS water solubility [56]. CS functionalized with various imidazole moieties (imidazole acetic acid, urocanic acid, histidine) was developed keeping in mind advantages for buffering capacity, better endosomal escape, and ultimately gene transfection [57]. Polymers with varying degrees of imidazole substitutions (5.6%, 12.9%, and 22.1% of the glucosamine) on CS were synthesized and the polyplexes formed upon DNA complexation were evaluated for β-galactosidase activity in 293T and HepG2 cells after transfection [58]. The polymer with highest substitution enhanced β-galactosidase expression. In another study, urocanic acid functionalized CS synthesized by EDC/NHS coupling reaction and complexed with DNA exhibited low cytotoxicity, high protection against nucleases, and better transfection efficiency compared with unfunctionalized CS [59]. It was also found that the transfection efficiency determined by luciferase activity in 293T cells improved as the degree of substitution increased. Histidine and CS were linked to cysteine acting as a linker by disulfide bonds [60]. Complexes prepared by histidine-modified CS with pDNA exhibited a broader buffering range, higher cellular uptake, and were more widely distributed in the cytosol suggesting the endosomal escape provided by histidine due to its high buffering capacity. The histidine-functionalized CS/pDNA nanoparticles also showed higher gene expression than that of unmodified CS/pDNA complexes, measured as luciferase activity in HEK293 cells. One of the studies utilized a multi-step approach that involved generation of deoxycholic acid conjugated CS followed by its complexation with siRNA in the first step [61]. The second step involved mixing of this complex in PLGA solution and emulsification in water to form nanoparticles, wherein deoxycholic-acid-conjugated CS/siRNA complex is stabilized within polymeric PLGA. These nanoparticles exhibited improved siRNA structural stability, efficient intracellular delivery, and sustained gene silencing activity in MDA-MB-435-GFP cells, as measured by GFP expression. In conclusion, the gene delivery efficiency of chitosan-based delivery vectors is dependent upon various factors, such as MW, degree of deacetylation, the charge ratio of chitosan to DNA/siRNA (N/P ratio), the chitosan salt form used, the DNA/siRNA concentration, pH, serum, additives, the preparation techniques of chitosan/nucleic acid particles, and the routes of administration.

### 3.3. Hyaluronic Acid-Based Vectors

Hyaluronic acid (HA) is composed of alternating disaccharide units of D-glucuronic acid and D-N-acetylglucosamine, which are linked via alternating β-1,4 and β-1,3 glycosidic bonds. Unlike chitosan, it is anionic polymer and has hydroxyl and carboxylic acid functional groups for chemical modifications to tune their physicochemical properties to achieve the expected drug-delivery goals [62]. It has been found that certain receptors, such as CD44 and CD168, which are overexpressed in cancer cells, are natural receptors of HA. Thus, HA can be used to facilitate selective adhesion and targeted drug delivery to these cells.

## 4. Polymer-Based NA Vehicles

Polymers have a broad application in the fields of electronics, conductors, and environmental technologies, and drug delivery has not been an exception. Within drug delivery, GT has benefited the most from polymeric delivery systems thanks to the chemically tunable nature of polymers [63]. Cationic polymers have been explored the most in nucleic acid delivery as they can associate with nucleic acids through electrostatic interactions to form polyplexes [64]. The cationic polymer must suffice several qualities for effective polyplex-mediated gene delivery [65]. Amongst physical properties, the polymer should be capable of condensing the nucleic acid payloads into particles of suitable dimensions that can help accumulation into the tissue of interest. Additionally, it should be able to protect the nucleic acid from the harsh environment in the body as well as any undesired interaction with biological components. It should facilitate cell specific targeting, endosomal escape, and delivery of the nucleic acid payload at the intended site of action within the cell. Furthermore, the polymer should be nontoxic, non-immunogenic, and biodegradable. A single polymer can rarely fulfil all these qualities and, as a result, additional functional elements may have to be included within the polyplex.

Nucleic acid binding is influenced by the chemical structure of the polymer (1) number of charged groups; (2) type of charged group (primary, secondary, and tertiary amines); (3) degree of branching in the polymer, to mention a few. In addition, factors such as ionic strength, concentration, N/P ratios, and the process of polyplex formation also influence the polymer affinity to nucleic acids [66]. The type of nucleic acid affects the choice of the polymer as different nucleic acids differ in their molecular weights, the number of charges, and the site of action. 

Cationic polyplexes—although attractive for in vitro and in vivo efficacy—suffer from drawbacks of relatively low transfection efficiency [67]. These early polycations have been explored to a great extent in literature and will not be expanded upon in this review.

The next generation of polycations were developed with a branched structure with the goal of complexing larger nucleic acid cargoes as well as showing enhanced transfection efficiency [68]. These types of polymers showed a better ability to escape endosome than early linear polycations. They are usually composed of multiple blocks of polycations that contain many proton-accepting groups, such as amines, and can achieve endosomal escape by causing an influx of chloride and water into the endosome upon increased protonation in the acidic endosomal environment. This results in bursting of endosome and subsequent escape [69]. PAMAM and PEI are commonly reported polycations that exhibit the proton sponge effect and are discussed in the following sections.

### 4.1. PAMAM-Based Vectors

PAMAM dendrimers are repetitively branched structures made up of amine and amide units [70]. The amine groups are modifiable and can form complexes with a variety of nucleic acid payloads through electrostatic interactions [71]. Additionally, they possess qualities such as high molecular uniformity, narrow molecular weight distribution, and they are biocompatible and water soluble [72]. All these factors lead to PAMAM dendrimers being efficient in the gene transfer of nucleic acid drugs. There have been many generations of PAMAM dendrimers, with superior gene transfer activity observed for higher generations [73]. However, increased cytotoxicity has also been observed as the generation increased, making dendrimers with lower generation and asymmetric structure useful, and displaying low cytotoxicity [74]. During the last decade, the gene delivery strategies using PAMAM can be classified as follows: (1) having no surface functionalization; (2) having a functionalized surface with PEG, antibodies, etc.; (3) being in conjugation with other delivery systems, such as liposomes and nanoparticles; (4) having supramolecular self-assembly nanoparticles.

PAMAM with no surface modification represents early development work which was more focused on exploring the utility of PAMAM as a gene delivery polymer [75]. The work focused on basic biophysical analysis and looking at the cell cytotoxicity and transfection [76]. In subsequent years, modifying the PAMAM terminal amine groups became more commonplace, and these changes were aimed at overcoming the limitations, such as toxicity and limited in vivo transfection efficiency [77]. In this section, we cover few recent examples of delivery systems that have leveraged modified PAMAM dendrimers and PAMAMs in conjugation with other systems, either as delivery vehicle or as a functionalization. A mechanistic study by Vasumathi and co-workers looked at free energy calculations and inherent structure determination using atomistic molecular dynamics [78]. The complexation behavior was studied using siRNA and two generations of PAMAM dendrimers (G3 and G4). An increase binding energy was observed with increasing generation possibly due to increased charge ratio.

Protection of nucleic acid from biological environment is a precursor to successful delivery [79]. Abdelhady and co-workers explored the ability of flexible and rigid PAMAMs to shield siRNA from RNase degradation using atomic force microscopy [80]. Both the dendrimers showed a time dependent degradation of siRNA. However, complexes formed at lower N/P ratio showed better resistance to siRNA degradation and reduced toxicity. An and co-workers synthesized a series of succinylated 4th generation zwitterionic PAMAM dendrimers and investigated the role of change on DNA packaging [81]. The assembly of DNA induced by these PAMAMs was investigated using small angle X-ray scattering (SAXS). They observed that higher degrees of succinylation reduced polymer–DNA interactions, resulting in less stable polyplexes. Lowering the pH increased the charge and resulted in tighter DNA packaging. This report showed that zwitterionic PAMAMs can be used to tune polymer–DNA interactions to improve DNA packaging and engineer gene delivery vectors. Additionally, the authors proposed that these PAMAMs would exhibit lower cytotoxicity due to reduced net charge and zwitterionic nature. 

With respect to cancer applications, overcoming multidrug resistance (MDR) remains one of the biggest challenges [82]. Li and co-workers synthesized PAMAM-MDR1 siRNA (siMDR1) polyplexes surface modified with h-R3 antibody, which showed reversal of MDR in breast cancer cell lines [83]. Moreover, these polyplexes worked synergistically with Paclitaxel, inhibiting tumor growth and induced cell apoptosis. Additionally, phospholipid-modified PAMAM has been shown to deliver siMDR1 to overcome MDR in human breast cancer cell lines MCF-7/ADR cells [84]. These hybrid nanoparticles could act as delivery vehicles for drug–nucleic acid or nucleic acid combinations.

As mentioned before, the latest iterations of PAMAM applications include hybrid applications where PAMAM is used in conjunction with other nanocarriers, such as nanoparticles, liposomes, etc., for delivering genetic material [85,86]. Nanoparticles modified with PEG have been shown to be promising nucleic-acid-delivery vehicles [87,88]. Lim and co-workers reported PAMAM–nanodiamond hybrid nanocomplexes with E6/E7 oncoprotein siRNA for treating human-papillomavirus-induced cervical cancer [89]. They showed that the nanocomplexes showed low cell cytotoxicity and significant tumor suppressing effect. Additionally, cellular uptake studies showed that PAMAM played a role in adjusting the pH to aid endosomal escape.

### 4.2. Polyethylenimine (PEI)-Based Vectors

PEI was one of the earliest vectors which showed impressive results during in vitro transfection experiments [90]. In 1997, Baker and co-workers reported PEI as a simple and inexpensive way of condensing plasmid DNA [91]. Based on the linkages between the repeating units, PEI can be branched (bPEI) or linear (lPEI) [92]. bPEI vectors have been used to deliver DNA, RNA, and ONs. High-molecular-weight bPEI up to 800 KDa has been used for gene-delivery applications [93]. The polyplexes formed were stable and showed higher transfection efficiency as compared with low-molecular-weight bPEIs. However, a corresponding decrease in cell viability was observed. This drawback could be overcome by using low molecular PEIs and increased the polymer concentration.

Although there have been a lot of reports on the use of PEI-based vectors for nucleic acid delivery, very few studies have focused on mechanistic aspects [94]. Gonzalez-Dominguez and co-workers studied the impact of physicochemical properties of DNA/PEI polyplexes on transient transfection of mammalian cells [95]. They investigated how the preparation time affects the physicochemical properties of the polyplexes and correlated it with the transfection efficiency. They used a 25 kDa lPEI at a 1:2 DNA: PEI *w*/*w* ratio and with a final DNA concentration of 1 µg/mL. The transfection was carried out in a serum-free-suspension-adapted HEK 293 cell line. They concluded that a strong relationship between DNA/PEI polyplex aggregation level and transfection efficiency existed. Aggregation did not affect the entire population evenly resulting in heterogenous complexes. Incubation time was a major determinant to be considered during polyplex preparation. Na and Cl concentrations were another parameter to be considered because remarkable levels of Na and Cl were detected in the particle, which probably came from the medium. Consequently, NaCl was indicated as a driving force for polyplex evolution in the medium as polyplexes prepared in water did not show the same transfection efficiency. SEM and TEM images also showed salt precipitates on the imaging grids. Surprisingly, the aggregates seemed to contribute significantly to transfection, probably as they carried more DNA and acted as reservoirs. Overall, this work took an interesting route towards understanding polyplex formation to improve reduce transfection variability and increasing production yield by manipulating simple parameters.

Based on the literature, PEI/DNA polyplexes have been repeatedly shown to be more stable than PEI/siRNA polyplexes [96]. Ziebarth and co-workers examined the potential causes of this difference by using molecular dynamic simulations to study complexation between PEI and nucleic acids [97]. They reported that both the types of polyplexes are stabilized by similar interactions. However, the number of interactions between PEI/DNA is greater than PEI/siRNA polyplexes with the interactions between protonated amines and DNA being particularly enhanced.

### 4.3. Synthetic Polymeric Vectors

The polymeric vectors discussed in the previous sections are not biodegradable and thus have no established route to leave the cells after administration. This extended exposure to foreign materials can lead to toxic effects. As a result, polymers that degrade to non-toxic or natural metabolites can enhance biocompatibility of the delivery system. Synthetic polycations can be biodegradable, enhancing their safety profile for systemic gene delivery.

Synthetic biodegradable polycations are generated by conjugating low-molecular-weight monomers into polymers using biodegradable or stimulus responsive linkers such as sulfide or ester bonds [98]. Most explored biodegradable polycations are PLA, PGA, and PLGA. They degrade via hydrolysis, thus are also sustained release materials. Significantly, PLGA has been approved by FDA [99]. PLGA-based delivery systems have been widely reported for nucleic acid payloads. Kalvanagh and co-workers reported preparation of IFN-λ plasmid DNA, encapsulated in PLGA nanoparticles [100]. They showed enhanced protection against DNase and increased gene delivery efficiency. Especially in delivery of siRNA, PLGA has shown a major promise compared with PEI and PAMAMs. Risnayanti and co-workers reported evaluation of PLGA nanoparticles to co-deliver MDR1 and BCl2 siRNA to treat ovarian cancer [101]. They hypothesized that the use of these particles would overcome drug resistance. Their results demonstrate enhanced cell killing in paclitaxel- and cisplatin-resistant ovarian cancer cell lines. Following similar approach, Su and co-workers reported therapeutic efficacy of PLGA nanoparticles in co-delivering Paclitaxel and stat3 siRNA using lung cancer cell line [102]. As a next step, the surface of the PLGA nanoparticles could be decorated with ligands to enable these particles to impart target specificity to these particles. Khan and co-workers decorated the surface of PLGA nanoparticles with N-acetylgalatosamine [103]. These particles were used to encapsulate survivin siRNA and assessed for therapeutic efficacy for treatment of hepatocellular carcinoma. In a mouse model, the nanoparticle-treated mice showed 75% improvement in weight loss and a significant reduction in tumor volume as compared with mice treated with free siRNA and untreated mice.

### 4.4. Pharmacologically Active Polycations

There have been multiple reports of pharmacologically active polycations, most of them utilizing a polycationic backbone and attaching small molecule drugs to create a polymeric drug [104]. Many groups have leveraged this strategy to attach small molecule drugs which contain primary or secondary amines to create pharmacologically active polycations that can complex nucleic acid payloads using charge interactions [105]. The resulting delivery systems can be utilized to deliver not only nucleic acid payloads but also can be designed to modulate the activity of the small molecule component. Kanvinde and co-workers reported synthesis of a polymeric chloroquine carrier [106,107]. By modifying the small molecule drug into a macromolecular structure, they were able to modify the pharmacokinetics to suit oral route as well as systemic of delivery [108,109]. Zhu and co-workers reported the synthesis and evaluation of self-immolative polycations that could act as both gene delivery vectors and prodrugs to target polyamine metabolism in cancer [110]. The synthesized polycation could degrade into small molecule drug Benspermine, that could normalize levels of various polyamines in cancer cell resulting in cell sensitization. Additionally, the polycation could complex TNFα DNA and induce cell death within the sensitized cancer cells. The enhanced anti-cancer efficacy of this system was demonstrated in multiple cancer cell lines. Using the same polymeric system, Xie and co-workers showed similar findings using miR-34a microRNA [111]. Xie and co-workers also reported synthesis of a polycation using a poly (amido amine) anti-CXCR4 drug and miR-200c microRNA [112]. This polymeric system was shown to inhibit the invasiveness of cholangiocarcinoma.

## 5. Lipid-Based NA Vehicles

Lipids can also be used to condense and encapsulate nucleic acid payloads [113]. The major benefits of lipids include their non-toxicity, ability for easy scale-up, and amenability to synthetic chemistry allowing for structural modification to bestow specificity and targeting [114]. In general, lipid structure is represented by the presence of a hydrophilic head and hydrophobic tail [115]. Moreover, the hydrophobic nature of lipids improves the payload integrity for extended periods of time [116,117]. Overall, development of lipid vectors can be pursued by three major routes which leverage their structural characteristics [118]

- Screening libraries of lipids to choose structurally effective for the application.
- Modification of currently existing lipid to enhance transfection efficiency.
- Develop new lipids to target specific target cells.

The selection of lipids based on the nanoparticulate systems is usually based on self-assembly of these lipids around the nucleic acid payload in solution [119]. Based on conformation in solution, lipids can be classified as cylindrical lipids, inverse conical lipids, and conical lipids [120]. Cylindrical lipids commonly form bilayers, conical lipids form inverted micellar structures, while inverse conical lipids form micelles. Self-assembly is also driven by modulating the polarity of the solution to harness the difference in polarity of lipid head and tail groups [121].

By either individual or a combination of these approaches and their self-assembly characteristics, various types of lipids have been developed. Various lipids have been used by themselves or in combination with other lipids to develop nanoparticulate gene-delivery systems. Table 4 shows the various lipid types being currently used in lipid-based vectors.

### 5.1. Liposomes

Liposomes are closed phospholipid vesicles with one or more concentric bilayers that form spontaneously in aqueous media and represent the earliest generation of lipid nanoparticles [122]. Lipid nanoparticles comprise solid lipid nanoparticles that have the cargo dispersed throughout the lipid core matrix, liposomes with single or multiple phospholipid bilayer architecture, or unilayered micellar phospholipid vesicles. Among these, liposomes represent the class of delivery systems with the highest extent of clinical translation, with nine liposome-based therapeutics having received approval worldwide by respective regulatory authorities [123]. Four clinical trials for LNP-based mRNA therapeutics and eight clinical trials for mRNA vaccines against cancers have been conducted [124].

Liposomes can efficiently encapsulate both hydrophilic and hydrophobic drugs and as a class have shown the most significant clinical potential in the delivery of small molecules for cancer therapy [125]. The biologically inert nature, high drug loading capacity, biocompatibility, and biodegradability of liposomes make them promising candidates for drug delivery vehicles in cancer treatment [126,127]. Liposomes can be subjected to surface functionalization using peptides, glycans, and antibodies to actively target moieties expressed homogenously and practically exclusively in cancer cell, which have shown a higher degree of internalization [128,129,130]. Breast-cancer-associated markers, such as HER2, have shown a 1000-fold higher expression in tumor cells than unaffected tissue [129,131]. Hepatic tumor cells have also been shown to express the transferrin receptors 2.780-fold higher compared with normal liver tissue [132]. Previous reports in the literature have reviewed the development of stealth liposomes that can evade macrophage uptake, also allowing for fine tuning of the metabolic fate of the cargo when delivered in vivo [129,133]. PEGylation of liposomes allows for evasion of macrophage uptake through reduced opsonization and preventing access to bilayer surface through steric hindrance [134,135]. Liposome can also be primed to release the nucleic acid cargo with inclusions imparting pH sensitivity, photosensitivity, or temperature sensitivity [136,137,138].

He and co-workers showed the therapeutic potential of liposomal CRISPR plasmid DNA coding for Cas9 and single-guide RNA targeting the ovarian-cancer-related DNA methyltransferase 1 (DNMT1) gene (gDNMT1) in a murine ovarian cancer model [139]. The study also reported lower adverse events with the CRISPR therapy than a parallel paclitaxel therapy, which further highlights the advantages of this approach. Zhang and co-workers reported inhibition of lung tumor growth in BALB/c nude xenografts upon liposomal administration of anti-CYP1A1 siRNA and leading to the downregulation of CYP1A1 mRNA [140]. In a proof-of-concept study, Moitra and co-workers developed an aptamer-guided, pH-sensitive liposomal formulation to delivery plasmid DNA as well as siRNA to EpCAM-overexpressing cancer stem cells [141]. Wang and co-workers developed transferrin-modified liposomes (Tf-PL) for the targeted delivery of acetylcholinesterase (AChE) gene, administered subcutaneously to treat liver cancer xenografts grafted in nude mice [132]. LErafAON is a liposome-encapsulated c-raf antisense oligonucleotide that reduces Raf-1 in tumor cells, making them better primed for radiotherapy [142]. Li and co-workers utilized a more intensive human cancer xenograft models of various origins in cynomolgus monkeys to evaluate the therapeutic efficacy and safety of a cationic liposomal formulation to deliver plasmid DNA coding for mature human neutrophil peptide-1 (HNP1), albeit with injection site toxicities and heightened immune activity [143]. 

### 5.2. Lipid Nanoparticles (LNPs)

Onpattro represents the first clinically approved cationic liposomal formulation encapsulating siRNA to sequester and silence transthyretin for treatment in hereditary transthyretin-mediated amyloidosis [144]. The first venture of lipid-based mRNA cancer therapy was reported by Conry and co-workers in 1995, with the administration of luciferase and mRNA encoding carcinoembryonic antigen as a cancer vaccine [145]. Hou and co-workers have reviewed and summarized the clinical trials addressing the utility of lipid-based vectors in the delivery of mRNA and siRNA as vaccines and as therapeutics [124]. The most common strategy for the mRNA vaccines comprises codelivery of antigen mRNA, and other RNA interference molecules to inhibit immune checkpoints [146,147]. siRNA has emerged as a promising platform to inhibit tumor growth. However, this application has been limited by the various drawbacks of this nucleic acid upon administration in vivo, such as rapid enzymatic degradation, sequestration and clearance by immune cells, and poor penetrance through cell membranes [148]. However, liposomal formulations encapsulating siRNAs have shown enhanced bioavailability, better pharmacokinetic parameters, and target specificity [149]. The most employed cationic lipid architecture for delivery of mRNA and siRNA comprises 3β-[*N*-(*N*′,*N*′-dimethylaminoethane)-carbamoyl] cholesterol (DC-Chol) or dimyristyloxypropyl-3-dimethyl hydroxyethyl ammonium (DMRIE), and dioleoyl phosphatidylethanolamine (DOPE) [150]. The development of ionizable cationic lipids, such as DODAP (1,2-dioleoyl-3-dimethylammonium propane), has allowed the liposomes to provide high loading of negatively charged nucleic acids since the larger size of the cargo and the inability to use transmembrane pH gradients for loading [123]. Other cationic lipids, such as DOTAP (1,2-dioleoyl-3-trimethylammonium-propane) and DOTMA (*N*-[1-(2,3-dioleoyloxy) propyl]-*N*,*N*,*N*-trimethylammonium methyl sulfate), have shown a high degree of encapsulation and formulation stability upon administration in vitro and in vivo, albeit with some issues reported regarding the poor release properties of these vesicles [149]. A better selection of neutral lipids such as 1,2-dioleoyl-sn-glycero-3-phosphatidylcholine (DOPC) has shown better results in the development of siRNA-based liposomal formulations for treatment of mouse xenograft tumor models [150,151,152,153]. This avenue did not show significant escalation in immune response, adverse events, or injection site toxicity upon repeated intravenous administrations, which is a common issue with viral vectors. 

### 5.3. Solid Lipid Nanoparticles

Solid lipid nanoparticles (SLN) are crystallized lipid structures with a solid lipid core and an amphiphilic exterior shell. The choice of lipid composition of the SLN influences the overall shape of morphology of the particle, where triglyceride based SLNs have a platelet shape, whereas monostearates have been shown to produce spherical particles [154,155]. In contrast with liposomes, SLNs care composed of solid lipids. The common approach used in the formulation of SLNs comprises lipid acids, such as mono/di/tri glycerides, or waxes stabilized by ionic or non-ionic surfactants [156]. The most used lipids include hydrogenated fatty acids such as Lubritab™, Dynasan™ P60, Sterotex™ HM, or Lipo™, stabilized by emulsifiers, such as Pluronic F 68 and F 127 [157]. While the utility of solid lipid nanoparticles (SLN) has been explored by some previous reports in the literature, they results have been mixed due to evident cytotoxicity of the cationic lipids [157,158].

While LNPs hold potential for development as nucleic acid vectors with many advantages, so far, the clinical success of LNPs has been overshadowed by viral vectors [159]. Botto and co-workers have demonstrated the therapeutic efficacy of cationic SLN-based formulations of plasmid DNA to achieve RNA interference of NUPR1-regulated genes to inhibit hepatocellular carcinoma in vitro [159]. A previous report by Mirahadi and co-workers have investigated the role and potential for SLNs in cancer diagnostics and theranostics [160].

## 6. Understanding the Regulatory Landscape and the Translational Elements

Despite the extensive research exploration on non-viral gene-delivery systems, there have been many challenges for successful commercial translation. One of the major reasons for this discrepancy is the limited understanding of process development [161]. This section touches on information from FDA guidance on Chemistry, Manufacturing, and Control (CMC) Information for Human Gene Therapy Investigational New Drug Applications (INDs).

Within the United States, gene therapies are subject to oversight by two major federal agencies within the Department of Health and Human Services: (i) the Food and Drug Administration (FDA), and (ii) the Office of Biotechnology Activities (OBA) at the National Institutes of Health (NIH). Gene therapy protocols are primarily reviewed and assessed by the Recombinant DNA Advisory Committee (RAC), organized by OBA. The FDA has the legal authority to regulate gene and cell therapy products under the Investigational New Drug (IND) application, the Biologics License Application (BLA), and the Investigational Device Exemption (IDE). These regulations are found in 21 CFR 312, 21 CFR 600 and 21 CFR 800, respectively. Cancer gene therapy (CGT) products are evaluated by the Office of Cellular, Tissue, and Gene Therapies (OCTGT) in the Center for Biologics Evaluation and Research (CBER). The application of current good manufacturing practices (cGMPs) is required under section Federal Food, Drug, and Cosmetic Act at all stages of clinical investigations. Recently, the FDA issued revised guidelines for chemistry manufacturing and control of advanced medical products, including gene and cell therapies. Important elements of current guidelines include detailed description of relevant critical quality attributes (CQAs) regarding safety and biological activity of the product as they are understood during the time of filing. It requires organizations to provide a detailed description of MOA of final products along with submission of information of raw materials, excipients, and intermediate process materials of the non-viral product. Details include key CMC elements, such as temporary storage of bulk harvest, concentration of drug products, steps in purification, sterilization, bioburden, final non-viral drug product, and fill–finish parameters. A robust analytical testing strategy is required for manufacturers to determine identity, purity, potency, and safety of the final product [162]. Additionally, companies are required to ensure the integrity of the final drug product throughout the strategic handling at clinical sites, from shipping, to storage, to stability indicating methods, to handling of the product at intermediate pharmacies, to dosage and administration at the clinic. While these guidelines may change from time to time, they aid to protect patients and provide transparency as the field continues to evolve to avoid some of the past missteps. Prior to initiating a phase 1 clinical trial, companies are required/encouraged to communicate with the FDA before submitting an IND for initiation of a non-viral clinical trial. A pre-IND meeting provides an opportunity for open dialogue between CBER/FDA and the company to discuss planned IND CMC and clinical content and obtain CBER/FDA advice to avoid any future potential clinical hold.

Similarly, in the European Union (EU), marketing authorizations are evaluated via a centralized procedure. The Committee for Advanced Therapeutics (CAT) and the Committee for Medicinal Products for Human Use (CHMP) are responsible for the validation and scientific evaluation for approval. The CAT evaluates the quality, efficacy, and safety of the gene therapies to prepare an opinion draft, which eventually supports the final decision made by CHMP. This marketing authorization via the centralized procedure may be granted in three ways: standard marketing authorization, conditional marketing authorization (when an innovative medicine addresses an unmet medical need yet a positive benefit–risk balance by sufficient clinical data is demonstrated), and marketing authorization under exceptional circumstances in those extreme situations where a disease is rare, or a clinical endpoint is difficult to measure. There are many parallels that exist between EMA and FDA, allowing companies to often target approvals in both the territories.

In the currently evolving field of non-viral vectors, significant process development is still required in design, manufacturing components, sterile drug product, aseptic processes, cold chain supply management, and many more areas. Development of advanced analytical technologies that enhance understanding of biophysical and biological attributes of non-viral quality attributes will be required for harmonization across the biopharmaceutical industry. Significant challenges in chemistry manufacturing and control strategy modes are still needed to be overcome to bring non-viral gene therapy to the mainstream, biologic-dominated market. This requires continuous improvements in understanding genome packaging in various non-viral vector systems by process and design engineering. Improvements in manufacturing cannot happen in isolation—it will require a consolidated effort with major companies to advance technologies in bulk substance titer control, chromatography growth, temperature control, sterile filtration, fill–finish technologies, and innovation in dosing. Finally, the regulatory landscape needs to reflect current knowledge and technologies to allow enough flexibility for the field to advance. This is particularly necessary for providing pharmaceutical scientists, academics, and clinicians ample opportunity to turn GT into mainstream medicine.

With the recent successes in multiple phases of ongoing clinical trials, breakthrough siRNA-LNP by Alnylam, and the commercial success of mRNA-LNPs with Pfizer and Moderna COVID vaccines, it seems likely that non-viral therapy will soon be included in the treatment armamentarium for various indications [163]. Table 5 highlights nucleic acid therapies for cancer that utilize non-viral vectors that are currently in clinical trials, along with their status, cancer type, and target gene. As with any drug for cancer therapy, gene therapies must meet the approval standard of being safe and effective. However, there are unique features and challenges for sponsors of gene therapies which must be addressed to continue exploration of non-viral vectors in gene delivery applications in cancer.

## 7. Conclusions

With the widespread exploration of delivery vectors for nucleic delivery, the modulation of structure, charge, toxicity, and specificity will continue to be guiding principles. The applications of non-viral systems have diversified by development of more sophisticated nucleic acid payloads, such as siRNA, mRNA, and microRNA, as well as gene editing tools. Additionally, with frequent redosing being a probable factor in cancer therapy, non-viral vectors have added benefits over viral vectors. Advances in combination therapy of cancer from a target specificity and pharmacokinetic perspective will continue to drive development of pharmacologically active polymers. These factors, in addition the multiple clinical and commercial products, are expected to result in newer generations of non-viral vectors which will be biodegradable, stimuli-responsive, target-specific, and safe. In combination with physical gene-delivery techniques, such as electroporation and sonoporation, these systems can usher in a new era of genetic medicine with enhanced target effects and reduced off-target toxicity.

## Figures and Tables

**Table 1:** Nucleic-acid-based therapeutics.

Characteristics	Plasmid DNA	siRNA	shRNA	miRNA	Antisense Oligonucleotide
Properties	Extra chromosomal circular DNA molecules.	Double-stranded RNA molecules, 20–25 bp in length.	Artificial RNA molecule with a tight hairpin turns.	Small noncoding RNA molecule (containing about 22 nucleotides).	Short strand of deoxyribonucleotide analog that hybridizes with the complementary mRNA.
Molecular weight	−0.5–5 kbp	19–22 bp	25–29 bp (stem) 4–23 nucleotides (loop)	21–24 nucleotides	18–21 nucleotides
Mechanism of action	Express specific gene. Replace faulty gene.	RNA interference by endonucleolytic cleavage of mRNA.	RNA interference by integration into host genome and transcribed to bind to RISC and cleave mRNA.	RNA interference by translational repression.	Induction of RNaseH enzyme. Steric hindrance of ribosomes causing translational arrest. Interfere with mRNA maturation or incorrect splicing in the nucleus.
Advantages	Can be used to express a missing gene. Relatively stable due to structure.	Can be synthesized and chemically modified for stability and specificity.	Required in lower amount, lower cost, and toxicity.	Less immunogenic than proteins, stable, can be synthesized and chemically modified.	No nuclear barrier: designing is easier.
Disadvantages	Need to enter nucleus to exert action. Insertional mutagenesis. Difficult to construct and formulate.	Poor PK parameters, low stability, off-target effects, transient effects.	Need viral vectors for delivery, overall high cost of development.	Cannot be used to express a gene of interest.	Nuclear barrier can exist for some therapeutic applications.
Immune response	TLR9	RNAs are generally recognized by three main types of immunoreceptors: TLR, protein kinase R, and helicases			TLR9 (if there are CpG motifs in the AON sequence).

**Table 2:** Advantages and limitations of viral vectors.

Viral Vector	Advantages	Limitations
Lentivirus	Effective for long period; Low host immune response.	8 kb size limitation for gene; Integrating; In vivo transfection is challenging; Safety concerns.
Retrovirus	Can transfect, difficult to transfect cells; Higher safety as advanced generations self-inactivating.	8 kb size limitation for gene; Integrating; Needs active cell transport; Safety concerns.
Adeno-associated virus	Long duration of in vivo expression; Non-integrating; Low immune response in host.	4.5 kb size limitation for gene; Potential safety concerns of insertional mutagenesis.
Adenovirus	High in vivo transfection efficiency; Non-integrating; Can transfect, difficult to transfect cells.	7.5 kb size limitation for gene; Repeat dosing impossible due to immune response; Short duration of in vivo transfection.
Herpes simplex virus	Effective for long period; Safe in immunocompromised individuals; Large insert possible (30 kb); Broad cell type application.	Large-scale production is challenging.

**Table 3:** Physical methods for delivering NAs.

Delivery Methods	Mechanism	Advantages	Limitations
Naked plasmid DNA	Administration via a syringe and needle into tissue of interest.	No carrier; Simplest and safest.	Low transfection efficiency due to rapid nucleic acid degradation in serum; Inapplicable for deep-tissue tumors.
Ballistic DNA (particle bombardment or gene gun)	Using high speeds achieved by high voltage, spark, or helium pressure discharge to deliver DNA-coated heavy metal particles across tissue membranes.	Precise delivery of DNA doses; Widely used for ovarian cancer applications.	Low penetration making application to deep-tissue tumors difficult.
Electroporation	Utilizing electrical gradient to achieve gene transfer across cell membrane.	Good efficiency and reproducibility.	Incorrect usage may result in tissue damage; Limited accessibility for internal organs.
Sonoporation (Ultrasound + microbubble)	Usage of ultrasound waves to permeabilize cell membrane and enhance DNA uptake; Used in conjunction with microbubbles to encapsulate nucleic acid.	Safety and flexibility.	Low transfection efficiency.
Photoporation	Utilizes laser pulse to generate transient pores in cell membrane for DNA transfer.	Claims to be similar to electroporation.	Lacks documented evidence.

**Table 4:** Types of lipids used in lipid-based nucleic-acid-delivery systems.

Type of Lipid	Examples of Lipids	Structural Characteristics	Role in Delivery Systems
Cationic lipids	DOTMA, DOTAP	2 tails, cylindrical conformation, forms bilayers, positive charged.	Forms the bilayer around the payload, major structural lipid in liposome.
Ionizable cationic lipids	DODMA, DLinDMA, DLinMC3DMA	2 tails, inverse micellar structures, pKa between 4–6, charged at acidic pH and neutral at physiological pH.	Forms micellar structures around nucleic acids in acidic solutions. Used in conjunction with cationic and structural lipids. Major structural lipid in LNPs.
Helper lipids	Cholesterol, Phosphatidylcholines	Rigid lipids.	Form anchoring regions between the structural lipids and help to stabilize the lipid layer.
PEG-lipids	PEG-attached cholines	Helper lipids anchored via chemical linkage to PEG.	Included to modulate the circulation time of the vector. Can be diffusible or persistent depending on the anchoring group.

**Table 5:** Non-viral vectors that are in clinical trials for cancer therapy.

Nucleic Acid	Vector	Sponsor	Disease	Target Gene	Clinical Trial	Status
siRNA	LNPs	Dicerna Pharmaceuticals	Hepatocellular carcinoma	MYC	NCT02314052	Terminated
	LNPs	Dicerna Pharmaceuticals	Hematological and solid tumors	MYC	NCT02110563	Terminated
	CD polymer	Calando Pharmaceuticals	Solid tumors	RRM2	NCT00689065	Terminated
	Gold NPs	Northwestern University	Glioblastoma	Bcl-2	NCT03020017	Completed
	LNPs	Alnylam Pharmaceuticals	Solid tumors	KSP and VEGF	NCT01158079	Completed
	Liposomes	Silence Therapeutics	Pancreatic cancer	PKN3	NCT01808638	Completed
	LNPs	National Cancer Institute	Liver cancer	PLK1	NCT01437007	Completed
	LNPs	Dicerna Pharmaceuticals	Solid tumors	MYC	NCT02110563	Terminated
	LNPs	University of Florida	Glioblastoma	TN-C	NCT04573140	Recruiting
	LNPs	Arbutus Biopharma Corp.	Neuroendocrine/Adrenal tumors	PLK1	NCT02191878	Completed
	Polymeric matrix	Silenseed Limited	Pancreatic cancer	KRAS	NCT01676259	Recruiting
	LNPs	Silence Therapeutics	Solid tumors	PKN3	NCT00938574	Completed
	LNPs	M.D. Anderson Cancer Center	Hepatocellular carcinoma, GI tumors	EphA2	NCT01591356	Active
miRNA	Liposomes	Mirna therapeutics	Advanced cancers	miR-34	NCT01829971	Terminated
	Minicells	Asbestos disease research foundation	Lung cancer	miR-16	NCT02369198	Completed
	LNPs	Moderna	Solid tumors, ovarian cancer	OX40L T cell	NCT03323398	Active
mRNA	LNPs	Moderna	Solid tumors and lymphoma	OX40L T cell	NCT03739931	Active
	LNPs	Moderna	Ovarian cancer	OX40L T cell	NCT03323398	Active
	Lipopolyplex	Stemirna therapeutics	Esophageal cancer	T cells	NCT03908671	Not yet recruiting
	LNPs	Moderna	Solid tumors	TAA	NCT03313778	Recruiting
	Liposomes	BioNtech SE	Stage IV melanoma	NY-ESO-1, MAGE-A3, tyrosinase, and TPTE	NCT04526899	Recruiting
	Liposomes	BioNTech SE	Prostate cancer	TAAs	NCT04382898	Recruiting
	Liposomes	University Medical Center Groningen and BioNTech SE	Ovarian cancer	TAAs	NCT04163094	Recruiting
	Lipid based particle	Ludwig Institute for Cancer Research, Boehringer Ingelheim, MedImmune, CureVac, PharmaJet	Lung cancer	MUC1, survivin, NY-ESO-1, 5T4, MAGE-C2, and MAGE-C1	NCT03164772	Completed
ssRNA	Polymeric carrier	CureVac	Melanoma, squamous cell carcinoma of skin, head, and neck or adenoid cystivc carcinoma	TLR7/8/RIG-1	NCT03291002	Active

Abbreviations: Myc—master regulator of cell cycle entry and proliferative metabolism; RRM2—ribonucleoside-diphosphate reductase subunit M2; Bcl2—B-cell lymphoma 2; KSP—kinesin spindle protein; VEGF—vascular endothelial growth factor; PKN3—protein kinase N3; PLK1—polo-like kinase 1; TN-C—tenascin-C; KRAS—Kirsten rat sarcoma viral oncogene homolog; EphA2—ephrin type-A receptor 2, miR—microRNA; TAA—tumor-associated antigens; NY-ESO-1—New York esophageal squamous cell carcinoma-1; MAGE—melanoma-associated antigen; TPTER—transmembrane phosphatase with tensin homology; MUC1—Mucin 1; 5T4—trophoblast glycoprotein; TLR—Toll-like receptor; RIG-1: retinoic-acid-inducible gene I.

## Data Availability

Not applicable.
